# Supplementary material for: An antiplasmid system drives antibiotic resistance gene integration in carbapenemase-producing Escherichia coli lineages
Source: Nat Commun. 2024 May 15;15:4093. doi: 10.1038/s41467-024-48219-y (PMC11096173; doi:10.1038/s41467-024-48219-y)
Supplement: Supplementary file 1 — Supplementary Information [file 41467_2024_48219_MOESM1_ESM.pdf]

Supplementary information for

An antiplasmid system drives antibiotic resistance gene integration in carbapenemase-producing *Escherichia coli* lineages

[1,2,3]Pengdbamba Dieudonné Zongo, [1,3,#]Nicolas Cabanel, [1,3,#]Guilhem Royer, [3,4]Florence Depardieu, [5]Alain Hartmann, [6,7,8]Thierry Naas, [1,3,&]Philippe Glaser,[1,3,&]Isabelle Rosinski-Chupin[*]

[1]Ecology and Evolution of Antibiotic Resistance Unit, Institut Pasteur, Paris, France; [2]Sorbonne Université, Paris, France ; [3]Université Paris Cité [4]; Synthetic Biology Unit, Institut Pasteur, Paris, France ; [5]UMR AgroEcologie 1347, INRAe, Université Bourgogne Franche-Comté, Dijon, France ; [6]Team Resist, INSERM UMR 1184, Université Paris-Saclay; [7]Department of Bacteriology-Hygiene, Bicêtre Hospital, APHP; [8]Associated French National Reference Center for Antibiotic Resistance, Le Kremlin-Bicêtre, France.
# and &: Contributed equally to this work

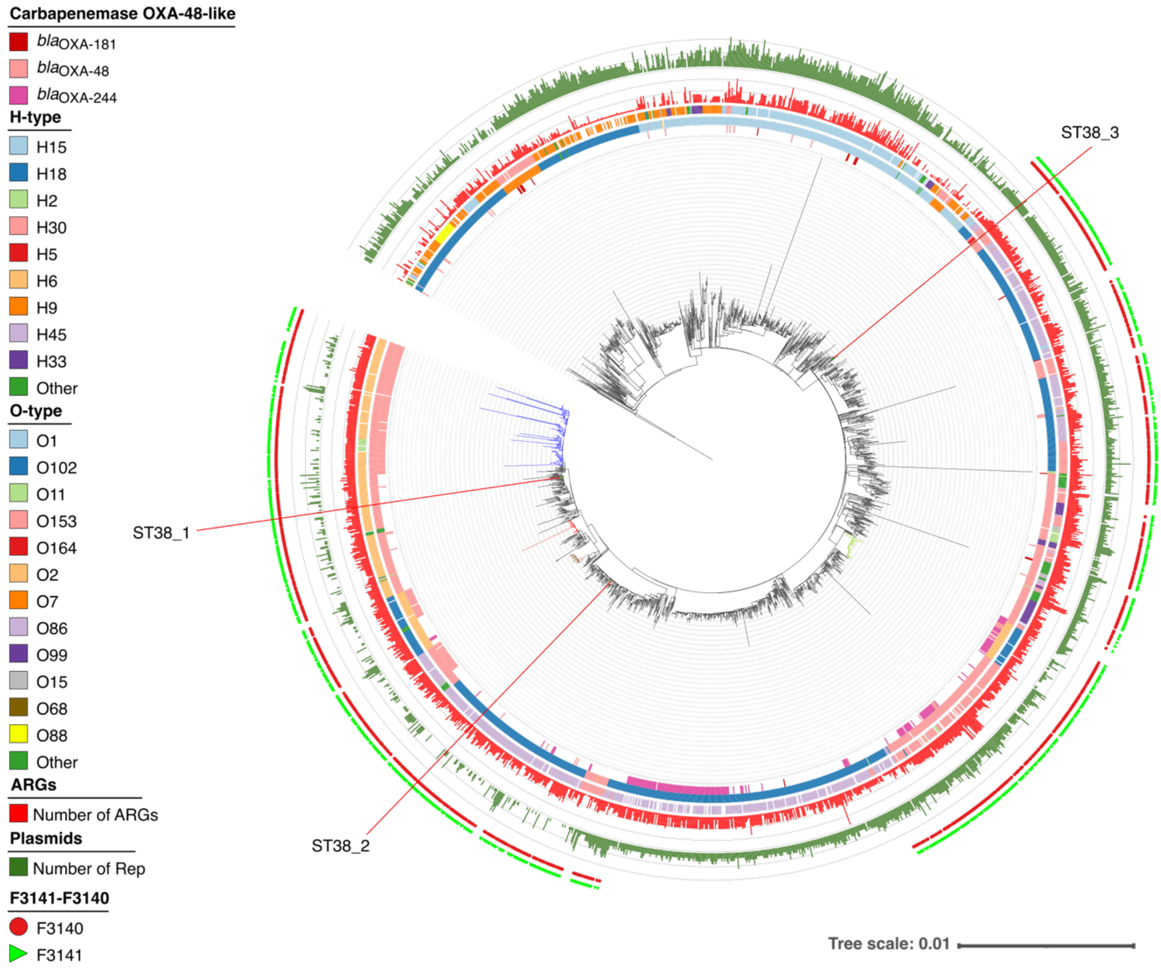

Supplementary Figure 1: Phylogeny of ST38 *E. coli*. Core genome phylogeny of a collection of 1907 *E. coli* ST38 strains from the NCBI database and Enterobase and the three strains of the study. The list of the strains and their characteristics are provided in Supplementary Data 10. The ST38 single locus variant ST963 strain CNRC6O47 was used as outgroup to root the maximum-Likelihood tree generated by RAxML[50] after removing regions of recombination by Gubbins[51]. Genomic features are indicated as in the figure key from the inside to the outside circles: carbapenemases OXA-48-like, H-type and O-type antigen, Numbers of ARGs as determined by using ResFinder[60], Number of plasmid replicon sequences (Rep) as determined by using PlasmidFinder[55] and presence of F3141-F3140 antiplasmid defence system. Worldwide disseminated chromosomally integrated lineages are coloured in blue, red, brown and light green. The recipient strains ST38_1, ST38_2 and ST38_3 are indicated with red solid lines.

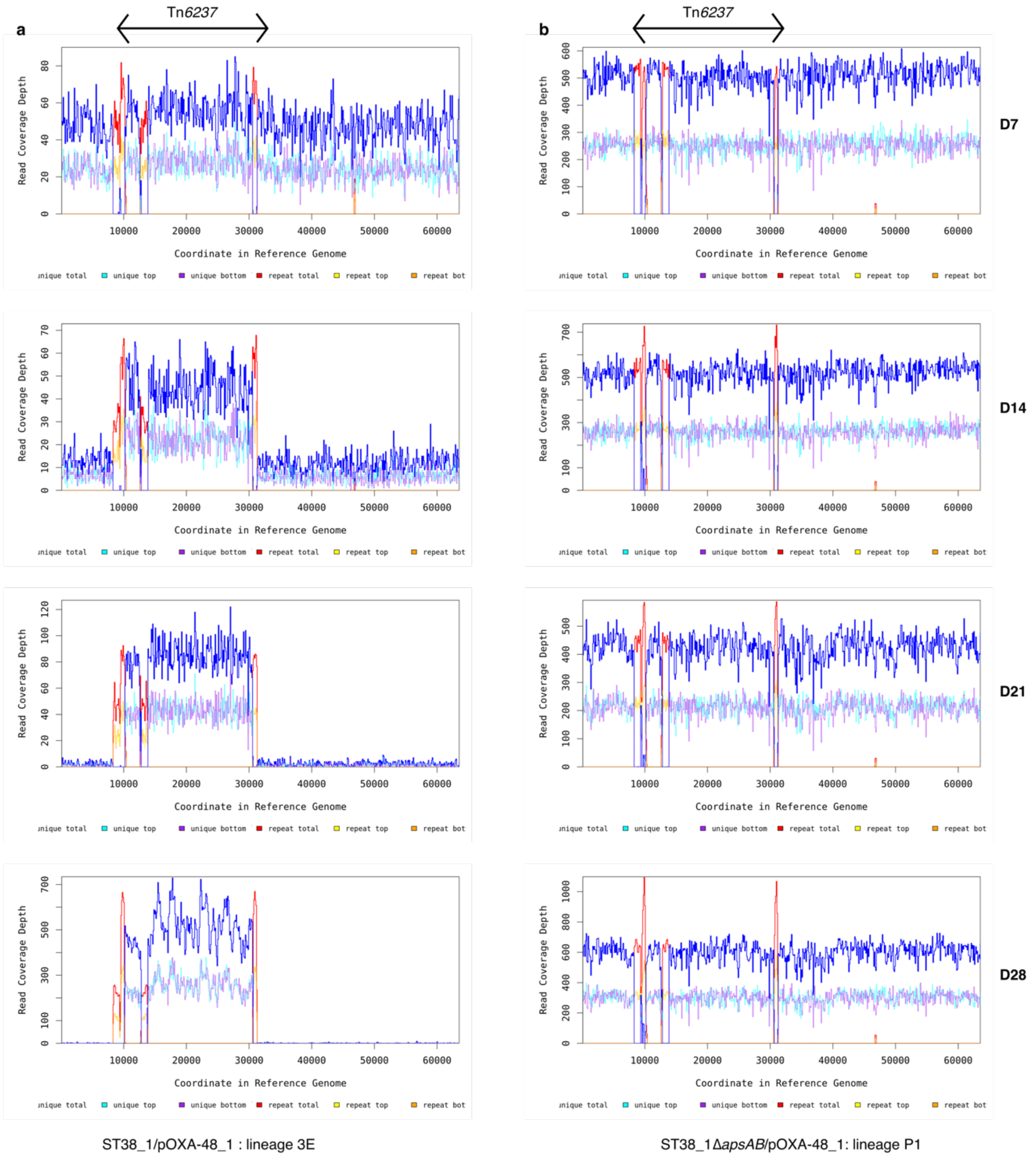

Supplementary Figure 2: pOXA-48_1 plasmid is gradually lost during experimental evolution of ST38_1 transconjugants. Evolution of pOXA-48_1 plasmid coverage during experimental evolution from day 7 to day 28, as obtained by Breseq[46] mapping of pool sequencing of an evolved population on pOXA-48_1 reference sequence. X-axis shows read-coverage depth and Y-axis pOXA48_1 coordinates. Tn*6237* is shown by double headed arrows. **a**. pOXA-48_1 coverage in ST38_1 (Lineage 3E). **b**. pOXA-48_1 coverage in ST38_1 Z103Δ*apsAB* (Lineage P1).

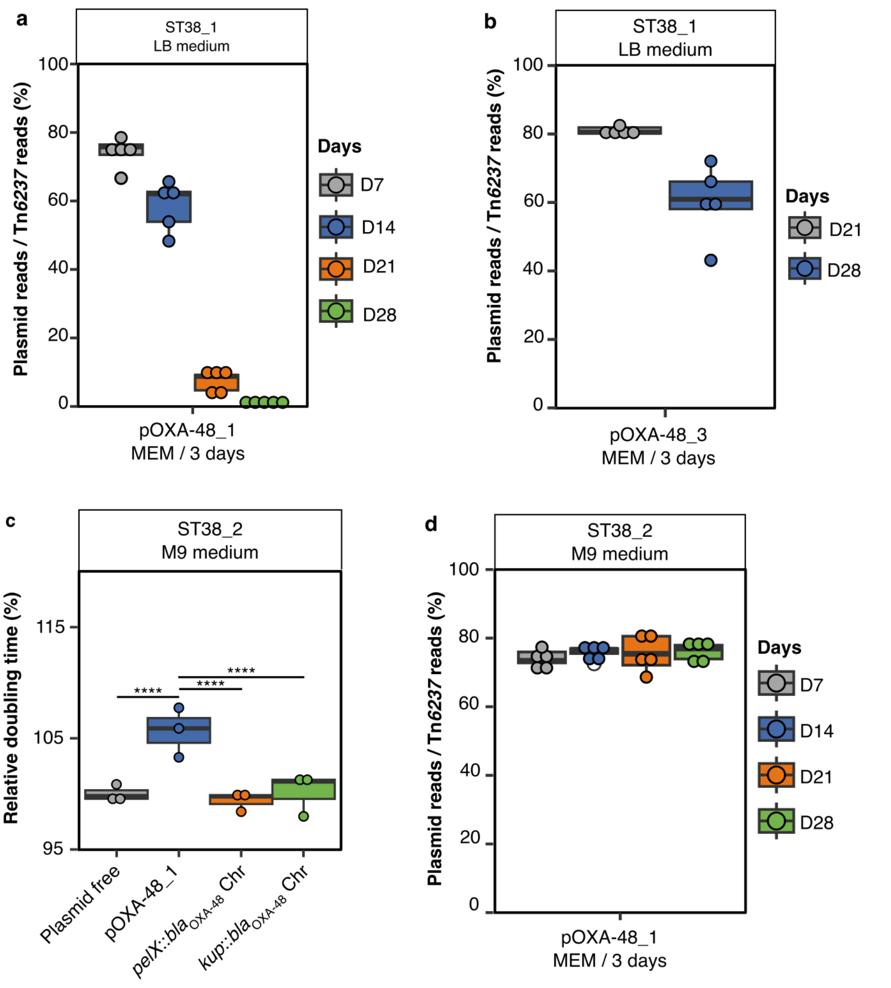

Supplementary Figure 3: Tn*6237* insertion is associated with pOXA-48 plasmid loss. a. b. and d. Quantification of plasmid loss by population WGS. Plasmid loss was estimated by calculating the ratio of the number of reads on three pOXA-48_1 regions of same size, two outside and one inside Tn*6237* respectively. **a**. pOXA-48_1 in ST38_1 evolved in LB medium is gradually lost from day 7 (D7) to day 28 (D28). **b**. pOXA-48_3 in ST38_1 transconjugants evolved in LB medium is partially lost at D28. **c**. Comparison of the relative doubling time of $bla_{\text{OXA-48}}$ integrants and the original transconjugant calculated at exponential growth phase in the absence of meropenem. The relative doubling time is calculated as the ratio of the doubling time to the doubling time of one plasmid-free strain. Chr for chromosome. **d**. pOXA-48_1 in ST38_2 evolved in M9 medium remains unchanged between D7 and D28. Figures a, b and d show the results from five independent lineages, Figure c from three biologically independent experiments. Boxplots show median, box bounds 25th and 75th quartiles, whisker bounds minimum and maximum excluding outliers and outliers are value > 1.5 × interquartile range. For c, normal distribution of data was assessed with Shapiro-Wilk normality test. Statistical analysis was performed by using a pairwise two sample two-sided t-test and *p*-values (Source data) were FDR-adjusted. ****p<0.0001. In c: n=3, p=0.000516, 0.000271, 0.000699 (from left to right). Source data are provided as a Source Data File.

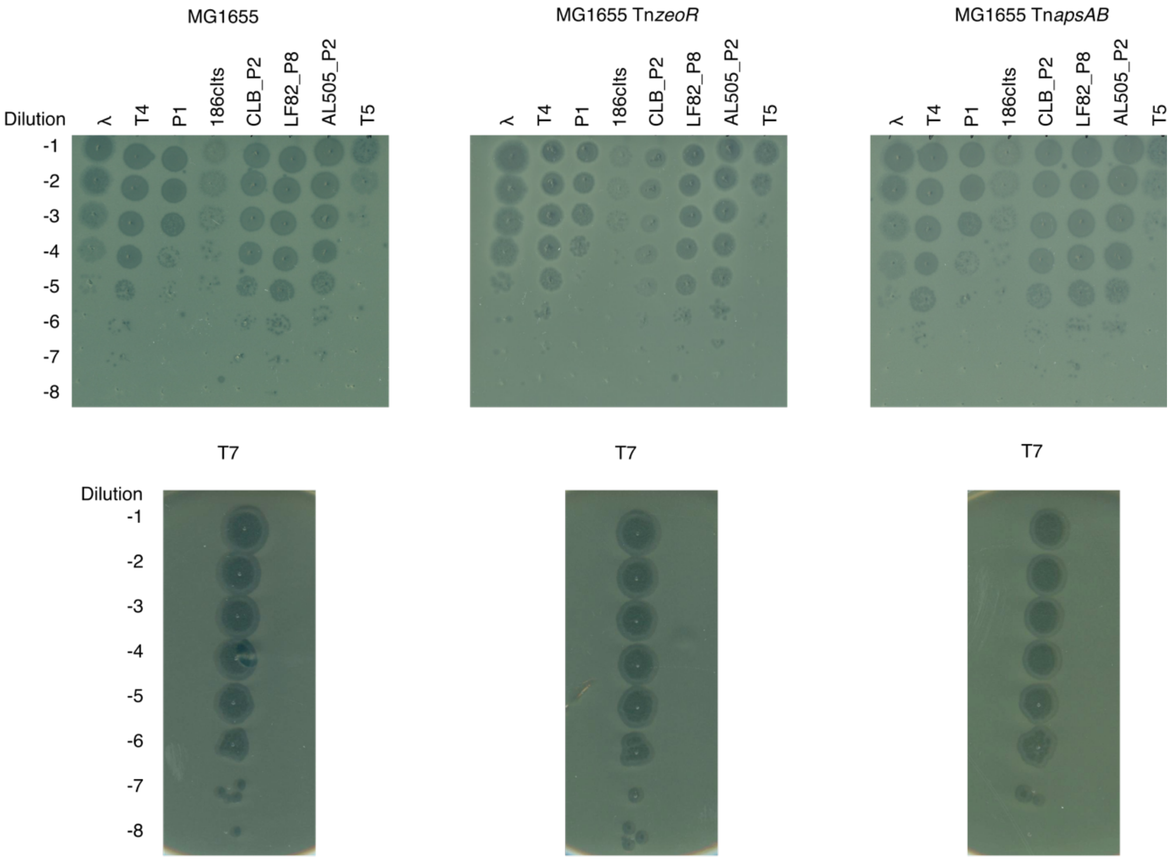

Supplementary Figure 4. ApsAB does not target eight tested phages. Serial dilutions of high titre lysates of phages lambda, T4, P1, 186cIts, CLB_P2, LF82_P8, AL505_P2, T5 and T7 spotted on MG1655 strain and its isogenic derivatives chromosomally carrying the miniTn*apsAB* (*apsAB* under the control of the arabinose inducible promoter p_{BAD}) or the miniTn*zeoR* as a negative control. Experiments were performed in three independent biological replicates and showed reproducible results. A representative experiment is shown. Dilution factor is indicated in Log10. Source data are provided at the end of this .pdf file.

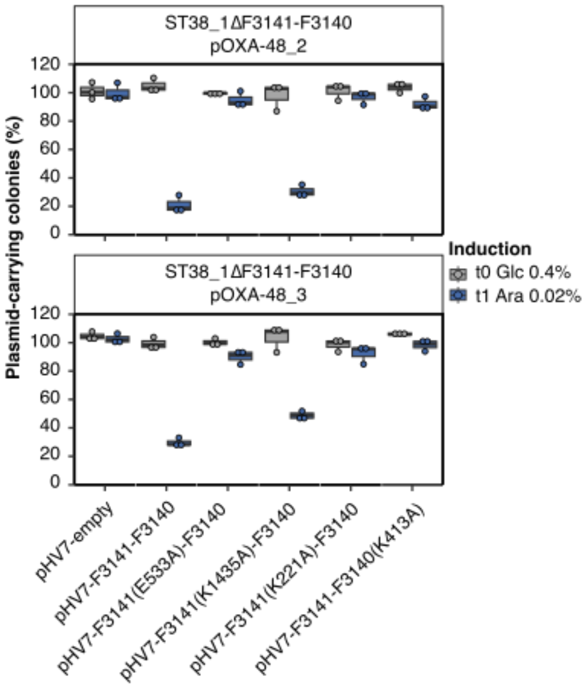

Supplementary Figure 5: Effect of mutations replacing key residues of the putative helicase (K221A, E533A) or nuclease (K1435A) domains of ApsA and of the predicted 5' nucleic acid binding domains (K413A) of ApsB on pOXA-48_2 and pOXA-48_3 maintenance. *apsAB* and variants were expressed under the control of the p_{BAD} inducible promotor in ST38_1ΔF3141-F3140 transconjugants. t0 refers to the value obtained following one 24h-passage in LB glucose (0.4%) and t1 to the value determined after two additional passages in LB arabinose (0.02%). pHV7-emty is a negative control. Figures show the results from three biologically independent experiments. Boxplots show median, box bounds 25th and 75th quartiles, whisker bounds minimum and maximum excluding outliers and outliers are value > 1.5 × interquartile range. Source data are provided as a Source Data File.

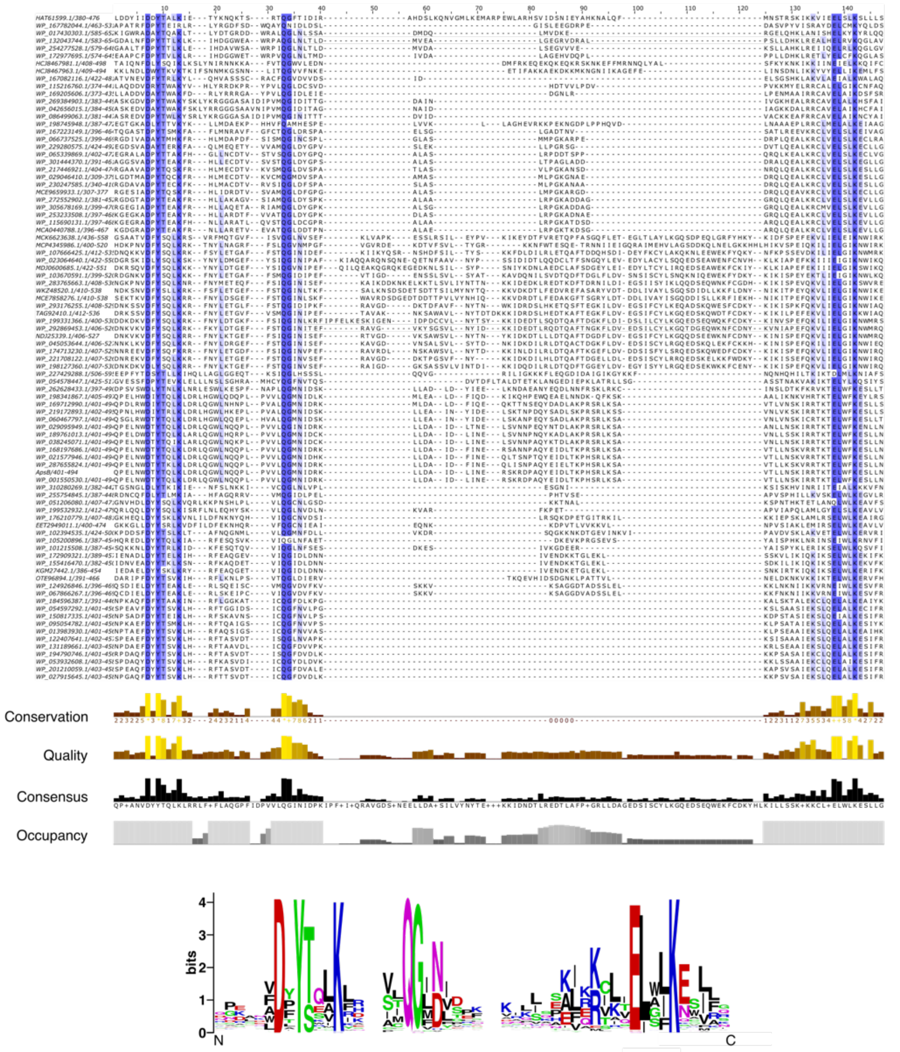

Supplementary Figure 6: Conserved residues in ApsB MID-like domain. Multiple alignment of ApsB-like proteins showed different conserved residues indicated in blue. The parameters Conservation, Quality, Consensus and Occupancy of amino acids are indicated below the alignment. The sequence logo generated by WebLogo [https://weblogo.berkeley.edu/logo.cgi] highlights the conserved residues. The list of ApsB-like proteins is provided in Supplementary Data 7.

Supplementary Table 1 : Meropenem MIC of plasmid-free strains, transconjugants and Tn*6237* integrants

MICs of strains before experimental evolution

Strains	[&]Plasmid	MIC meropenem µg/mL[#]
ST38_1	-	0.023
ST38_1 transconjugant Z103	pOXA-48_1	0.5
ST38_1 transconjugant Z112	pOXA-48_2	0.5
ST38_1 transconjugant Z122	pOXA-48_3	0.5
ST38_2	-	0.032
ST38_2 transconjugant Z3	pOXA-48_1	0.25
ST38_2 transconjugant Z41	pOXA-48_2	0.25
ST38_2 transconjugant Z62	pOXA-48_3	0.25
ST38_3	-	0.047
ST38_3 transconjugant Z142	pOXA-48_1	0.38
ST38_3 transconjugant Z147	pOXA-48_2	0.38
ST38_3 transconjugant Z152	pOXA-48_3	0.38

&: a dash indicates the recipient strain not carrying a pOXA-48 plasmid

MICs of strains with Tn*6237* chromosomal integration (after experimental evolution)

Strains	Exprimental evolution	MIC meropenem µg/mL[#]	Complementary informations
3E-p12	ST38_1/pOXA-48_1	0.25	Tn*6237* integration in IncF plasmid in *FIEDEJGE_04811*
5A-p14	ST38_1/pOXA-48_1	0.25	Tn*6237* integration between FIEDEJGE_02264 and *gadW*. One mutation in *malT*
5B-p16	ST38_1/pOXA-48_1	0.25	Tn*6237* integration in IncF plasmid in *yhcR*
N2_p45	ST38_2/pOXA-48_1	0.5*	Tn*6237* integration in *kup* + IS*1* insertion in *ompF* promotor (position -56)
N5_p48	ST38_2/pOXA-48_1	0.5*	Tn*6237* integration in *pelX* + Mutation R78W in *envZ*

#: Determined by E-test. Three biologically independent experiments were performed for each strain with reproducible results.
*: MIC of ST38_2 integrated strains is twice the one of the original transconjugant due to mutations in *ompF* and *envZ* genes involved in bacterial outer membrane permeability.

Supplementary Table 2 : Summary of the different evolution experiments.

<table>
<tr><th>Evolution experiment</th><th>Recipient strain</th><th>Plasmid [&]</th><th>Strain [#]</th><th>Medium</th><th>Condition [*]</th><th>Lineages</th></tr>
<tr><td colspan="7">Experimental evolution 1 (Fig. 2)</td></tr>
<tr><td>ST38_1/LB/No MEM</td><td rowspan="3">ST38_1</td><td>-</td><td>ST38_1</td><td>LB medium</td><td>No MEM</td><td>1A,1B,1C,1D,1E</td></tr>
<tr><td>ST38_1/POXA-48_1/LB/MEM/day</td><td>pOXA-48_1</td><td>Z103</td><td>LB medium</td><td>MEM / day</td><td>3A,3B,3C,3D,3E</td></tr>
<tr><td>ST38_1/POXA-48_1/LB/MEM/3 days</td><td>pOXA-48_1</td><td>Z103</td><td>LB medium</td><td>MEM / 3 days</td><td>5A,5B,5C,5D,5E</td></tr>
<tr><td colspan="7">Experimental evolution 2 (Fig. 3)</td></tr>
<tr><td>ST38_1/LB/No MEM</td><td rowspan="4">ST38_1</td><td>-</td><td>ST38_1</td><td>LB medium</td><td>No MEM</td><td>A1,A2,A3,A4,A5</td></tr>
<tr><td>ST38_1/POXA-48_1/LB/MEM/3 days</td><td>pOXA-48_1</td><td>Z103</td><td>LB medium</td><td>MEM / 3 days</td><td>B1,B2,B3,B4,B5</td></tr>
<tr><td>ST38_1/POXA-48_2/LB/MEM/3 days</td><td>pOXA-48_2</td><td>Z112</td><td>LB medium</td><td>MEM / 3 days</td><td>C1,C2,C3,C4,C5</td></tr>
<tr><td>ST38_1/POXA-48_3/LB/MEM/3 days</td><td>pOXA-48_3</td><td>Z122</td><td>LB medium</td><td>MEM / 3 days</td><td>D1,D2,D3,D4,D5</td></tr>
<tr><td colspan="7">Experimental evolution 3 (Fig. 3)</td></tr>
<tr><td>ST38_2/LB/No MEM</td><td rowspan="3">ST38_2</td><td>-</td><td>ST38_2</td><td>LB medium</td><td>No MEM</td><td>2A,2B,2C,2D,2E</td></tr>
<tr><td>ST38_2/POXA-48_1/LB/MEM/day</td><td>pOXA-48_1</td><td>Z5</td><td>LB medium</td><td>MEM / day</td><td>4A,4B,4C,4D,4E</td></tr>
<tr><td>ST38_2/POXA-48_1/LB/MEM/3 days</td><td>pOXA-48_1</td><td>Z5</td><td>LB medium</td><td>MEM / 3 days</td><td>6A,6B,6C,6D,6E</td></tr>
<tr><td>ST38_3/LB/No MEM</td><td rowspan="2">ST38_3</td><td>-</td><td>ST38_3</td><td>LB medium</td><td>No MEM</td><td>E1,E2,E3,E4,E5</td></tr>
<tr><td>ST38_3/POXA-48_1/LB/MEM/3 days</td><td>pOXA-48_1</td><td>Z142</td><td>LB medium</td><td>MEM / 3 days</td><td>F1,F2,F3,F4,F5</td></tr>
<tr><td colspan="7">Experimental evolution 4 (Fig. 3)</td></tr>
<tr><td>ST38_2/M9/No MEM</td><td rowspan="2">ST38_2</td><td>-</td><td>ST38_2</td><td>M9 medium</td><td>No MEM</td><td>M1,M2,M3,M4,M5</td></tr>
<tr><td>ST38_2/POXA-48_1/M9/MEM/3 days</td><td>pOXA-48_1</td><td>Z3</td><td>M9 medium</td><td>MEM / 3 days</td><td>N1,N2,N3,N4,N5</td></tr>
<tr><td colspan="7">Experimental evolution 5 (Fig. 6)</td></tr>
<tr><td>ST38_1/LB/No MEM</td><td rowspan="2">ST38_1</td><td>-</td><td>ST38_1</td><td>LB medium</td><td>No MEM</td><td>O1,O2,O3,O4,O5</td></tr>
<tr><td>ST38_1ΔapsAB/POXA-48_1/LB/MEM/3 days</td><td>pOXA-48_1</td><td>Z103ΔapsAB</td><td>LB medium</td><td>MEM / 3 days</td><td>P1,P2,P3,P4,P5</td></tr>
</table>

&: a dash indicates the parental strain not carrying a pOXA-48 plasmid
#: Refers to the ancestor of evolved lineages. See Supplementary Data 1.
*: MEM: meropenem at 0.1 $\mu g.ml^{-1}$.

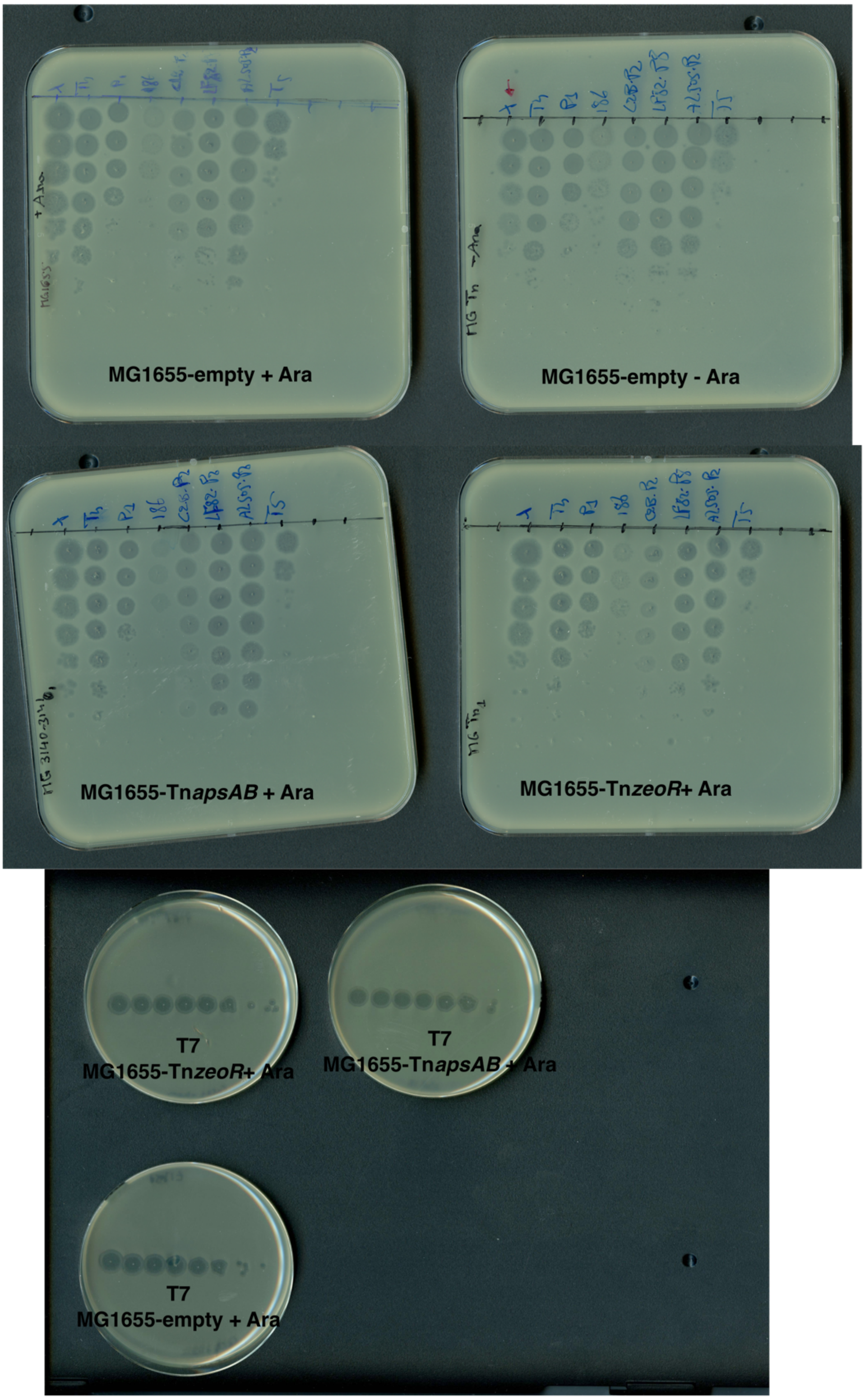

Source data for Supplementary Figure 4
