# Supplementary material for: An antiplasmid system drives antibiotic resistance gene integration in carbapenemase-producing Escherichia coli lineages
Source: Nat Commun. 2024 May 15;15:4093. doi: 10.1038/s41467-024-48219-y (PMC11096173; doi:10.1038/s41467-024-48219-y)
Supplement: Supplementary file 3 — Description of Additional Supplementary Files [file 41467_2024_48219_MOESM3_ESM.pdf]

<u>Description of Additional Supplementary Files</u>

File Name: Supplementary Data 1

Description: Bacterial strains and bacteriophages used in the study (Excel file).

File Name: Supplementary Data 2

Description: Proportions of *repA*$^-$ colonies in each evolved lineage after selection on LB plates + MEM (Excel file).

File Name: Supplementary Data 3

Description: Tn*6237*-insertion-sites in isolated colonies after evolution experiment (Excel file).

File Name: Supplementary Data 4

Description: IS*1* new junctions corresponding to candidate Tn*6237*- insertion sites (Excel file).

File Name: Supplementary Data 5

Description: Mutations detected in isolated colonies at day 28 of experimental evolutions (Excel file).

File Name: Supplementary Data 6

Description: Main characteristics of the sequenced bacteria isolated at day 28 of experimental evolutions (Excel file).

File Name: Supplementary Data 7

Description: *apsB* homologs retrieved by using PSI-BLAST on NCBI clustered nr database (Excel file).

File Name: Supplementary Data 8

Description: Identification of apsA homologs by using PSI-BLAST analysis on NCBI clustered nr database (Excel file).

File Name: Supplementary Data 9

Description: Plasmids and primers used in this study (Excel file).

File Name: Supplementary Data 10

Description: Main characteristics of the genome sequences used for the phylogenetic reconstruction of *E. coli* ST38 (Excel file).
