# Supplementary material for: An antiplasmid system drives antibiotic resistance gene integration in carbapenemase-producing Escherichia coli lineages
Source: Nat Commun. 2024 May 15;15:4093. doi: 10.1038/s41467-024-48219-y (PMC11096173; doi:10.1038/s41467-024-48219-y)
Supplement: Supplementary file 14 — Reporting Summary [file 41467_2024_48219_MOESM14_ESM.pdf]

nature portfolio

Corresponding author(s): Isabelle Rosinski-Chupin

Last updated by author(s): Apr 7, 2024

Reporting Summary

Nature Portfolio wishes to improve the reproducibility of the work that we publish. This form provides structure for consistency and transparency in reporting. For further information on Nature Portfolio policies, see our Editorial Policies and the Editorial Policy Checklist.

Statistics

For all statistical analyses, confirm that the following items are present in the figure legend, table legend, main text, or Methods section.

n/a	Confirmed	
☐	☒	The exact sample size (*n*) for each experimental group/condition, given as a discrete number and unit of measurement
☐	☒	A statement on whether measurements were taken from distinct samples or whether the same sample was measured repeatedly
☐	☒	The statistical test(s) used AND whether they are one- or two-sided<br>*Only common tests should be described solely by name; describe more complex techniques in the Methods section.*
☒	☐	A description of all covariates tested
☐	☒	A description of any assumptions or corrections, such as tests of normality and adjustment for multiple comparisons
☐	☒	A full description of the statistical parameters including central tendency (e.g. means) or other basic estimates (e.g. regression coefficient) AND variation (e.g. standard deviation) or associated estimates of uncertainty (e.g. confidence intervals)
☐	☒	For null hypothesis testing, the test statistic (e.g. *F*, *t*, *r*) with confidence intervals, effect sizes, degrees of freedom and *P* value noted<br>*Give P values as exact values whenever suitable.*
☒	☐	For Bayesian analysis, information on the choice of priors and Markov chain Monte Carlo settings
☒	☐	For hierarchical and complex designs, identification of the appropriate level for tests and full reporting of outcomes
☒	☐	Estimates of effect sizes (e.g. Cohen's *d*, Pearson's *r*), indicating how they were calculated

Our web collection on statistics for biologists contains articles on many of the points above.

Software and code

Policy information about availability of computer code

Data collection	-Commercial software: i-control 2.0.10 for Tecan infinite M Nano; guppy 5.0.14<br>Guppy 5.0.14 used with MinION Mk1C device
Data analysis	Open access softwares: -hybridSPAdes v 3.15.5 ;Canu 2.2 ;Circlator 1.5.5, SPAdes 3.15.5 , Breseq 0.35.7 , IGV 2.11.9, RStudio (R 3.6.3), GenomicAlignments package 1.22.1, R (version 3.6.3), MuscleW 3.8.31, Jalview 2.11.3, Alphafold2 implemented in Neurosnap (https://neurosnap.ai/) , Foldseek (https://search.foldseek.com/search), Pymol 2.5.5 , PSI_BLAST on https://blast.ncbi.nlm.nih.gov/, HHpred server (https://toolkit.tuebingen.mpg.de/tools/hhpred), GCsnap 1.0.17, SPAdes 3.12.0, QUAST 2.2, Parsnp 1.5.4, RAxML 8.2.12, Gubbins,Prokka 1.14.5, Abricate 1.0.1,,PlasmidFinder 2.1.1, web version of Clinker (https://cagecat.bioinformatics.nl/tools/clinker), iTOL v6 (https://itol.embl.de/], DefenseFinder (https://defensefinder.mdmlab.fr/)

For manuscripts utilizing custom algorithms or software that are central to the research but not yet described in published literature, software must be made available to editors and reviewers. We strongly encourage code deposition in a community repository (e.g. GitHub). See the Nature Portfolio guidelines for submitting code & software for further information.

Data

Policy information about availability of data

All manuscripts must include a data availability statement. This statement should provide the following information, where applicable:

- Accession codes, unique identifiers, or web links for publicly available datasets
- A description of any restrictions on data availability
- For clinical datasets or third party data, please ensure that the statement adheres to our policy

The data that support the findings of this study are provided within the manuscript and the associated supplementary materials. Full details and links to the publicly available databases [https://enterobase.warwick.ac.uk/], [https://defensefinder.mdmlab.fr/], [https://www.uniprot.org/], [https://www.ncbi.nlm.nih.gov/Structure/icn3d/], [https://www.ncbi.nlm.nih.gov/] used in bioinformatic analyses are provided in the methods and their associated references. Complete genome sequences of ST38_1, ST38_2 and ST38_3 and of the three pOXA-48 plasmids have been deposited at DDBJ/EMBL/GenBank (BioProject PRJEB71895). The corresponding Genome_ID are provided in Supplementary Table 1 (for ST38 strains) and 11 (for pOXA-48s). Illumina reads for the three K. pneumoniae used as donors of pOXA-48 have also been deposited at DDBJ/EMBL/GenBank (BioProject PRJEB71895) and their accession numbers are given in Supplementary Table 1. Sequence data from individual colonies and pools are available from the corresponding author upon request. Source Data are provided with this paper.

Research involving human participants, their data, or biological material

Policy information about studies with human participants or human data. See also policy information about sex, gender (identity/presentation), and sexual orientation and race, ethnicity and racism.

Reporting on sex and gender	N/A
Reporting on race, ethnicity, or other socially relevant groupings	N/A
Population characteristics	N/A
Recruitment	N/A
Ethics oversight	N/A

Note that full information on the approval of the study protocol must also be provided in the manuscript.

Field-specific reporting

Please select the one below that is the best fit for your research. If you are not sure, read the appropriate sections before making your selection.

☒ Life sciences ☐ Behavioural & social sciences ☐ Ecological, evolutionary & environmental sciences

For a reference copy of the document with all sections, see nature.com/documents/nr-reporting-summary-flat.pdf

Life sciences study design

All studies must disclose on these points even when the disclosure is negative.

Sample size	All experimental evolution experiments were performed on five independent lineages with equivalent results. The number of evolved lineages was based on standard protocols in the field. All other experiments were performed independently three times based on standard protocols in the field. No statistical methods was used to predetermine sample size.
Data exclusions	No data were excluded from the analysis.
Replication	All experimental data are representative of at least three biologically independent experiments.
Randomization	Individual colonies were randomly chosen for biological replicates.
Blinding	Blinding was not relevant to this study, since no manual counting or scoring was perfomed to obtain data.

Reporting for specific materials, systems and methods

We require information from authors about some types of materials, experimental systems and methods used in many studies. Here, indicate whether each material, system or method listed is relevant to your study. If you are not sure if a list item applies to your research, read the appropriate section before selecting a response.

Materials & experimental systems

n/a	Involved in the study
☒	☐ Antibodies
☒	☐ Eukaryotic cell lines
☒	☐ Palaeontology and archaeology
☒	☐ Animals and other organisms
☒	☐ Clinical data
☒	☐ Dual use research of concern
☒	☐ Plants

Methods

n/a	Involved in the study
☒	☐ ChIP-seq
☒	☐ Flow cytometry
☒	☐ MRI-based neuroimaging

Plants

Seed stocks	N/A
Novel plant genotypes	N/A
Authentication	N/A
